# A prospective case-control study to investigate retinal microvascular changes in acute dengue infection

**DOI:** 10.1038/srep17183

**Published:** 2015-11-25

**Authors:** Petrina Tan, David C. Lye, Tun Kuan Yeo, Carol Y. Cheung, Tun-Linn Thein, Joshua G. Wong, Rupesh Agrawal, Ling-Jun Li, Tien-Yin Wong, Victor C. Gan, Yee-Sin Leo, Stephen C. Teoh

**Affiliations:** 1 National Healthcare Group Eye Institute, Tan Tock Seng Hospital, Singapore; 2 Institute of Infectious Diseases and Epidemiology, Communicable Diseases Centre, Tan Tock Seng Hospital, Singapore; 3 Singapore Eye Research Institute, Singapore National Eye Centre, Singapore; 4 Ophthalmology and Visual Sciences Academic Clinical Programme, Duke-NUS Graduate Medical School, National University of Singapore, Singapore; 5 Eagle Eye Center, Singapore; 6 Yong Loo Lin School of Medicine, National University of Singapore; 7 Saw Swee Hock School of Public Health, National University of Singapore

## Abstract

Dengue infection can affect the microcirculation by direct viral infection or activation of inflammation. We aimed to determine whether measured retinal vascular parameters were associated with acute dengue infection. Patients with acute dengue were recruited from Communicable Diseases Center, Singapore and age-gender-ethnicity matched healthy controls were selected from a population-based study. Retinal photographs were taken on recruitment and convalescence. A spectrum of quantitative retinal microvascular parameters (retinal vascular caliber, fractal dimension, tortuosity and branching angle) was measured using a semi-automated computer-based program. (Singapore I Vessel Assessment, version 3.0). We included 62 dengue patients and 127 controls. Dengue cases were more likely to have wider retinal arteriolar and venular calibers (158.3 μm vs 144.3 μm, p < 0.001; 227.7 μm vs 212.8 μm, p < 0.001; respectively), higher arteriolar and venular fractal dimensions (1.271 vs 1.249, p = 0.002; 1.268 vs. 1.230, p < 0.001, respectively), higher arteriolar and venular tortuosity (0.730 vs 0.546 [x10^4^], p < 0.001; 0.849 vs 0.658 [x10^4^], p < 0.001; respectively), compared to controls. Resolution of acute dengue coincided with decrease in retinal vascular calibers and venular fractal dimension. Dengue patients have altered microvascular network in the retina; these changes may reflect pathophysiological processes in the immune system.

Dengue is one of the most important mosquito-borne viral diseases in the world. Estimation of the global incidence of dengue infections per year is close to 400 million^1^. Locally, Singapore experienced an unprecedented epidemic in 2013, with 22,170 cases and eight deaths. The epidemic has continued into 2014, with more than 18,000 cases^2^.

Although the majority of symptomatic infections result in a relatively benign disease course, a small proportion of patients may develop severe disease. The pathogenesis of this disease has been linked to the ability of the dengue virus to infect immune, dendritic and endothelial cells^3^, giving rise to increased capillary permeability and subsequently biochemical and hemodynamic changes. Severe forms of dengue infection affect the microcirculation, as demonstrated by dengue virus directly infecting endothelial cells or activating inflammatory markers, such as interleukin-6 (IL-6) and interleukin-8 and tumor necrosis factor alpha^4^.

The retina offers a unique and easily accessible “window” to study the health and disease of the human microcirculation. There is increasing evidence to show that retinal microvascular abnormalities (such as retinal venular widening, higher retinal vascular fractal dimension and more tortuous retinal vessels) reflecting early changes of the structure and function of systemic small vessels are markers of systemic diseases and its risk factors^5^,^6^,^7^,^8^,^9^,^10^. Retinal microvascular changes in infectious diseases have been relatively understudied. However previous studies on cardiovascular risk factors and inflammation found evidence that retinal venular caliber may be influenced by systemic inflammation. Larger retinal venular calibers were associated with systemic markers of inflammation (high-sensitivity C-reactive protein (CRP) and IL6) and endothelial dysfunction (plasminogen activator inhibitor and soluble intercellular adhesion molecule)^11^,^12^

In this study, we aimed to first determine whether quantitatively measured retinal microvascular parameters were associated with dengue infection, compared with age-gender-race-matched healthy controls. We then further compared retinal microvascular parameters in patients with dengue between the acute and convalescent stages, and correlated retinal microvascular parameters with hematological and biochemical markers. We hypothesize that the systemic inflammation and endothelial dysfunction caused by dengue infection can trigger changes in the systemic microcirculation, which in turn are reflected as microvascular alterations in the retina.

## Methods

### Study population

This is a prospective case-control study conducted at the Communicable Diseases Center, Singapore between September 2011 and June 2012. Patients were recruited upon confirmation of their dengue status. Dengue was confirmed by real-time reverse transcriptase polymerase chain reaction or non-structural 1 (NS1) antigen. Probable dengue cases required fulfilling World Health Organisation (WHO) diagnostic criteria in the presence of positive rapid dengue IgM or IgG serology^13^. 127 age-gender-ethnicity matched normal healthy controls without fever were selected from the Singapore Prospective Study Program and Singapore Cardiovascular Cohort Study 2. The methodology of this population-based study is described in detail elsewhere^12^. Informed consent was obtained from all patients after explanation of the nature and possible consequences of the study. All study protocols were performed in accordance with the Declaration of Helsinki revised in 1989 and approved by the Institutional Review Board of the National Healthcare Group, Singapore (DSRB/A/2011/01796).

### Data collection

Patients’ demographic data, concomitant medical co-morbidities and laboratory results (hemoglobin, platelet count, total white cell count, liver function test, creatinine) were collected.

### Definitions of dengue severity

The definitions for dengue severity used in this study were based on the WHO 2009 dengue case definitions^14^. This is divided into dengue without warning signs, dengue with warning signs (abdominal pain or tenderness, persistent vomiting, clinical fluid accumulation, mucosal bleeding, lethargy, liver enlargement and hematocrit increase with rapid platelet drop) and severe dengue (severe plasma leakage, severe bleeding or severe organ impairment).

### Retinal photography

Study cases underwent 2 visits: at presentation and at convalescence (4 weeks after recruitment) with retinal photographs taken at both visits. The method for obtaining digital fundal photographs was described in previous studies^5^. Digital fundus photography was performed using a 45° retinal camera (Canon CR-DGi; Canon, Tokyo, Japan) with a digital camera back (10D SLR; Canon). Only the optic disc centered image was used for assessment.

### Quantitative measurements of retinal microvasculature

We used a semi-automated computer-assisted program (Singapore I Vessel Assessment [SIVA], version 3.0; National University of Singapore) to quantitatively measure a range of retinal vascular parameters (vascular caliber, vascular tortuosity, fractal dimension and branching angles) from digital photographs according to a standardized protocol.

**Figure 1:**
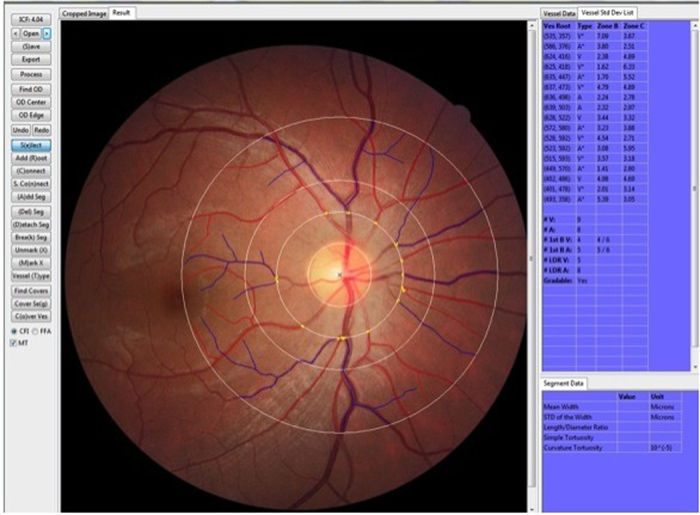
Retinal fundus photograph assessed quantitatively by the Singapore I Vessel Assessment (SIVA) software (version 3.0, National University of Singapore, Singapore) in a dengue patient. Arterioles are in red and venules are in blue. The measured area of retinal vascular parameters (caliber, fractal dimension, tortuosity and branching angle) was standardized as the region from 0.5 to 2.0 disc diameters away from the disc margin.

The measured area of retinal vascular branching, retinal vascular fractal dimension, and retinal vascular tortuosity was standardized and defined as the region from 0.5 to 2.0 disc diameters away from the disc margin (Fig. 1). Trained graders followed a standardized protocol and performed visual evaluations of the automated measurements. The graders were masked to the subjects’ identity and other measured parameters in the Singapore Advanced Imaging Laboratory Research. Inter- and intra-observer reliability measurements of the trained graders were previously performed^15^.

*Retinal vascular caliber.* Retinal vascular caliber was measured following a standardized protocol, based on the revised Knudtson-Parr-Hubbard formula, as described in other publications^16^. A pair of indices, the central retinal arteriolar and venular equivalents (CRAE and CRVE), summarizing the average arteriolar and venular calibers for each eye, was then calculated.

*Retinal vascular fractal dimension*. Fractal dimension was calculated from the skeletonized line tracing using a boxcounting method; a “global” measure summarizing the whole branching pattern of the retinal vascular tree^17^,^18^. Larger values indicated a more complex branching pattern.

*Retinal vascular tortuosity*. Retinal vascular tortuosity is defined as the integral of the curvature square along the path of the vessel, normalized by the total path length^19^,^20^. A smaller tortuosity value indicates a straighter vessel. The estimates were summarized as retinal arteriolar tortuosity and retinal venular tortuosity, representing the average tortuosity of arterioles and venules of the eye, respectively.

*Retinal vascular branching.* Retinal branching angle is defined as the first angle subtended between two daughter vessels at each vascular bifurcation^17^.

### Statistical analysis

Retinal vascular parameters were compared between patients with dengue and normal controls. Changes in retinal microvasculature were compared in patients with dengue between acute and convalescent visits. These differences were tested using the independent-samples t test and paired t tests for continuous variables, and the Pearon’s chi-squared test for categorical variables.

Ordinary least square (OLS) regression is sensitive towards outliers and influential observations. To overcome this problem, robust regression was performed to determine relationships between retinal vascular parameters and hematological or biochemical markers. Robust regression^21^ first fits the OLS regression and excludes influential observations. Huber and Biweights are used to assign weights to absolute residuals. The general idea is to downweigh large residuals. The standard error estimate uses the pseudovalues method. Hence, this method is robust toward outliers and non-normality yet does not compromise efficiency^21^,^22^, Logistic regression was used to ascertain associations of retinal vascular parameters with severe disease and warning signs. Analyses were performed with Stata 13.0 (Stata Corporation, Texas, U.S.A.) at a 5% significance level.

## Results

### Patient demographic information

A total of 62 dengue patients were recruited in the study. Patient demographic data and laboratory results at baseline and convalescence are shown in Table 1. The mean age was 33 years. Majority were males (87%) and of Chinese ethnicity (74.0%). Thirty-six patients (58%) had warning signs and 7 patients (11%) developed severe disease. There were significant changes in laboratory values in the patients from recruitment to convalescence. There were significant increases in platelet count (115 × 10^9^/L to 217 × 10^9^/L), total white cell count (3.43 × 10^9^/L to 5.90 × 10^9^/L), neutrophils (49.7% to 55.6%) and albumin level (40.1 g/L to 41.7 g/L). There were significant decreases in hemoglobin level (14.9 g/dL to 13.9 g/dL) and creatinine level (73.2 to 68.8). Liver transaminases also significantly normalized with aspartate aminotransferases decreasing from 77.9 U/L to 27.0 U/L and alanine aminotransferases decreasing from 74.4 U/L to 31.4 U/L. Significant decreases were found in hemoglobin level (14.9 g/dL to 13.9 g/dL) and creatinine level (73.2 μmol/L to 68.8 μmol/L).

### Retinal vascular parameters in dengue patients vs matched healthy controls

Comparisons between retinal vascular parameters in dengue patients at baseline and normal controls are shown in Table 2. Dengue patients were more likely to have a wider CRAE (158 μm vs 144 μm, p < 0.001), wider CRVE (228 μm vs 213 μm, p < 0.001), higher arteriole fractal dimension (1.271 vs 1.249, p < 0.05), higher venule fractal dimension (1.268 vs 1.230, p < 0.001), increased arteriolar tortuosity (0.730 × 10^4^ vs 0.546 × 10^4^, p < 0.001) and increased venular tortuosity (0.849 × 10^4^ vs 0.658 × 10^4^, p < 0.001). There was no difference in arteriole or venule branching angles between patients and controls.

### Retinal vascular parameters at enrolment vs convalescence in dengue patients

**Figure 2:**
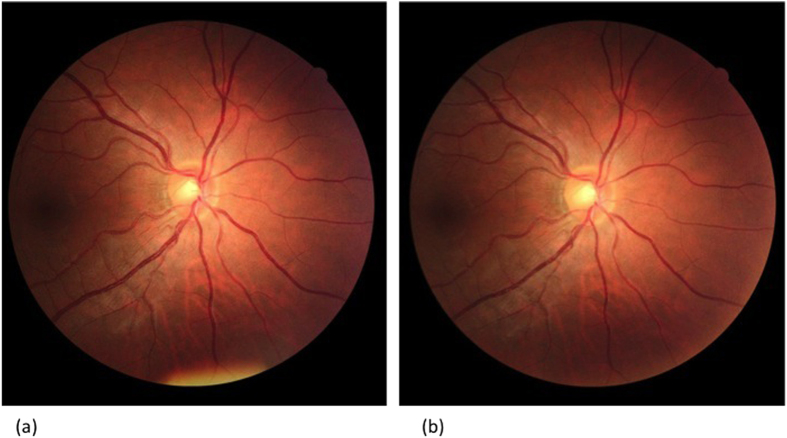
Example of a dengue patient (**a**) at acute dengue infection stage, and (**b**) at convalescence stage. The retinal arteriolar caliber (127.0 vs. 138.8 μm) and venular caliber (205.1 vs. 228.7 μm) are decreased at covalescence stage.

Resolution of infection coincided with changes of some retinal vascular parameters as shown in Table 3 and Fig. 2. Comparing parameters at enrolment to those at convalescence showed significant reduction of CRAE (p = 0.001), CRVE (p < 0.001) and venular fractal dimension (p = 0.007). The changes in arteriolar fractal dimension, vessel tortuosity and vascular branching angles were not significant.

### Hematological and biochemical parameters

Aspartate transaminase (AST) was noted to be associated with CRVE, where for every unit increment in AST, CRVE reduced by 0.407 μm (95% CI: −0.798, −0.017) for the dengue-adjusted (DA) and 0.430 μm (−0.810, −0.040) for the dengue and retinal-adjusted (DRA) model. Hemoglobin (DA: 0.01 [0.005, 0.016]; DRA: 0.010 [0.005, 0.016] and hematocrit (DA: 0.03 [0.016, 0.045]; DRA: 0.029 [0.014, 0.043] were also found to be positively associated with venular fractal dimension. Creatinine was found to be positively associated with venular fractal dimension for the DA model (0.072 [0.006, 0.138]) but not the DRA model (0.072 [−0.003, 0.147]). No associations with retinal vascular parameters were found for platelets, mean corpuscular volume (MCV), leucocytes, lymphocytes, neutrophils, alanine aminotransferase (ALT), urea, albumin or protein levels. These results are shown in Table 4.

### Dengue outcomes

None of the 4 retinal vascular parameters were significantly associated with severe disease or warning signs. (Table 5)

## Discussion

Our study set out to investigate the effects of dengue infection on retinal microvasculature and found that patients with acute dengue had significant differences in retinal vascular calibers, retinal vascular fractal dimensions and vascular tortuosity compared with matched healthy controls. Resolution of acute dengue infection resulted in significant reversal of such changes. There were correlations of liver transaminases and other biochemical parameters with venular caliber and fractal dimension respectively. To our best knowledge, our novel findings of retinal microvasculature changes in patients with acute dengue infection have not been previously reported.

The pathogenesis of systemic dengue infections is currently believed to be multifactorial, complex and not yet fully understood. Factors postulated in its pathogenesis include direct viral invasion or both humoral and cell-mediated immune responses. The severity of the infection is influenced by viral genotypes and host determinants^23^. An immune pathogenesis is likely involved given the natural history of dengue eye disease^24^ and resolution after steroid treatment in selected cases^25^. We do not believe that the retinal vasculature changes are secondary to a febrile illness, as we have previously published retinal vascular changes in afebrile patients with human immunodeficiency virus^26^.

*In vitro* data and autopsy studies suggest that three organ systems play an important role in the pathogenesis of dengue hemorrhagic fever or dengue shock syndrome: the immune system, the liver and endothelial cell linings of blood vessels^3^. Postulations of dengue infected endothelial cells contributing to pathogenesis include: increasing the viral load, secreting inflammatory mediators of cytokines, modulating complement pathways, or transforming the endothelium into an immunologic target of cellular and humoral immune responses^3^,^27^. The resulting immune–mediated reactions give rise to endothelial dysfunction and capillary permeability.

It has been proposed that the circulation of high levels of secreted NS1 in the presence of pre-existing heterologous non-neutralizing antibody may mediate complement activation and trigger plasma leakage^28^. In addition, it is believed that secreted NS1 from infected cells can activate the complement factors present in the fluid phase directly giving rise to further inflammation^29^. Our study findings of changes in retinal vascular calibers associated with systemic inflammation echoes previous studies on rheumatoid patients. Increased retinal venular calibers have been described in patients with autoimmune rheumatic diseases^30^ or rheumatoid arthritis^31^ compared to other hospital controls. Okada *et al.* also found that patients with autoimmune rheumatic diseases and elevated CRP levels had wider retinal venules than those with low or normal CRP^30^.

We postulate that the above mechanisms may account for suboptimal microcirculatory conditions during the acute phase of the dengue infection. The microcirculation thus responds with changes in microvasculature with dilated vessels and increased complexity of the microvasculature. However with recovery from the dengue infection and resolution of systemic inflammation, our results further showed that the microvasculature changes were reversible. Future *in vitro* studies will be required to correlate the pathophysiological changes with retinal microvasculature abnormalities.

Our study found associations between AST levels and CRVE. The liver is commonly involved in dengue infection in humans and mouse models^32^. Aminotransferase levels were found to increase in conjunction with dengue severity in a local study^33^. Significant elevation of liver aminotransferases were also found in cases of dengue warning signs and severe dengue as compared to cases without warning signs in a study on children from Bangkok^34^. In our study, AST was negatively associated with CRVE with increasing AST levels associated with narrower venular calibers. This is a useful finding and suggests that CRVE values may be associated with disease severity. There is promise that measurements of venular calibers via retinal photography may be a useful non-invasive tool in disease severity monitoring.

Hemoglobin, hematocrit and creatinine levels were associated with venular fractal dimension. We postulate that dengue patients with capillary leakage may suffer from hypovolemia. The resulting hypoxic and hypovolemic environment may give rise to a suboptimal microcirculation manifested as changes in fractal dimension.

There are a few limitations to our study. The study population is small but we have attempted to control this by comparing with age-gender-ethnicity matched normal controls. Due to the relatively young age of our dengue cohort, only one patient had cardiovascular co-morbidities of hypertension and hyperlipideamia. Although cardiovascular co-morbidities can affect retinal vasculature, we did not control for this. However in view of the small number, it is unlikely to change the results of the analysis. The small sample size might restrict our ability to demonstrate a significant association retinal vascular changes and severe forms of dengue. Despite the standardized protocols used, the retinal vasculature grading includes measurement errors related to subjective grader input and variability in image quality.

In summary, this is the first paper published on retinal microvasculature changes in patients with acute dengue infection. These changes are reversible with resolution of dengue infection and are promising non-invasive avenues of monitoring disease severity. It will be interesting to know if retinal vasculature changes predict severe forms of dengue and whether the changes persist in patients with severe disease. Larger prospective studies should be conducted for these purposes.

## Additional Information

**How to cite this article**: Tan, P. *et al.* A prospective case-control study to investigate retinal microvascular changes in acute dengue infection. *Sci. Rep.* **5**, 17183; doi: 10.1038/srep17183 (2015).

## Figures and Tables

**Table 1:** Characteristics of control (n = 127) and dengue patients (n = 62).

Characteristics	Controls	Dengue patients at baseline	Dengue patients at convalescence visit	P-value*
Age (years)	34.33 (7.19)	33.31 (8.53)		0.483
Gender (%)				
Males	111 (87)	54 (87)		0.777
Females	16 (12.6)	8 (13)		
Ethnicity (%)				
Chinese	96 (75.6)	46 (74.19)		0.995
Malay	4 (3.1)	2 (3.23)		
Indians	27 (21. 3)	14 (2.58)		
Mean arterial pressure	87.92 (7.47)	87.35 (8.97)	–	0.670
(mmHg)	–		86.60 (8.77)	0.487
Platelets (10^9^/L)		115.11 (51.18)	216.56 (55.17)	**<0.001**
Hemoglobin (g/dL)		14.86 (1.16)	13.91 (1.13)	**<0.001**
Total white blood cells (10^9^/L)		3.43 (1.46)	5.90 (1.46)	**<0.001**
Neutrophils (%)		49.70 (13.00)	55.62 (8.38)	**0.001**
Lymphocytes (%)		34.17 (9.46)	32.71 (7.50)	0.266
Alanine aminotransferases (U/L)		74.37 (129.61)	31.42 (15.33)	**0.009**
Aspartate aminothransferases (U/L)		77.89 (95.89)	27.00 (11.87)	**<0.001**
Creatinine (umol/L)	89.31 (13.91)	73.15 (13.74)	68.79 (12.90)	**<0.001 <0.001**
Albumin (g/L)		40.06 (2.75)	41.73 (2.34)	**<0.001**
Total protein (g/L)		68.97 (4.78)	70.13 (3.83)	0.051

^*^Paired t-test between baseline and convalescence visit.

Data presented are mean (standard deviation) or frequency (percentage) where appropriate.

**Table 2:** Changes in retinal vascular parameters between dengue patients at acute visit and controls.

Retinal vascular parameters	Dengue patients (n = 62)	Controls (n = 127)	*P-value
CRAE, μm	158.3 (13.14)	144.3 (9.76)	**<0.001**
CRVE, μm	227.7 (21.39)	212.8 (14.31)	**<0.001**
Arteriolar fractal dimension	1.271(0.048)	1.249 (0.044)	**0.002**
Venular fractal dimension	1.268 (0.053)	1.229 (0.043)	**<0.001**
Arteriolar tortuosity (x10^4^)	0.730 (0.137)	0.546 (0.116)	**<0.001**
Venular tortuosity (x10^4^)	0.849 (0.133).	0.658 (0.113)	**<0.001**
Arteriolar branching angle, deg	80.76 (7.85)	81.09 (9.01)	0.802
Venular branching angle, deg	79.86 (8.66)	79.19 (9.91)	0.647

^*^2-sample t-test between acute visit of dengue patients and controls. Values are presented as mean (standard deviation).

**Table 3:** Retinal vascular parameters in dengue patients at acute and convalescence visits (n = 62).

Retinal vascular parameters	Acute	Convalescence	P-value*
CRAE, μm	158.5 (13.23)	153.57 (12.34)	**0.001**
CRVE, μm	228.0 (21.39)	220.0 (16.87)	**<0.001**
Arteriolar fractal dimension	1.271 (0.048)	1.126 (0.046)	0.063
Venular fractal dimension	1.268 (0.053)	1.254 (0.045)	**0.007**
Arteriolar tortuosity (x10^4^)	0.730 (0.14)	0.740 (0.12)	0.242
Venular tortuosity (x10^4^)	0.849 (0.13)	0.854 (0.12)	0.931
Arteriolar branching angle, deg	80.76 (7.85)	80.36 (10.23)	0.596

^*^Paired t-test between acute and convalescent visit. Values are presented as mean (standard deviation).

**Table 4:** Relationship between laboratory parameters with retinal vascular parameters.

Blood parameters	Retinal vascular parameter	Dengue-adjusted estimates (DA)♀	Dengue & retinal-adjusted estimates (DRA)ψ
Aspartate aminotransferase (U/L)	CRVE	−0.41 (−0.798, −0.017)*	−0.43 (−0.81, −0.04)*
Hematocrit (%)	Venule fractal dimension	0.03 (0.016, 0.045)*	0.029 (0.014, 0.043)*
Hemoglobin (g/dL)	Venule fractal dimension	0.01 (0.005, 0.016)*	0.01 (0.005, 0.016)*
Creatinine (μmol/L)	Venule fractal dimension	0.072 (0.006, 0.138)*	0.072 (−0.003,0.147)

*Significant at 5% level.

^♀^Adjusted for absence or presence of fever.

**Table 5:** Association between retinal vascular parameters with dengue outcomes.

	Severe disease		Warning signs	
	aOR	95% CI	aOR	95% CI
Venular fractal dimension	0.99	0.98–1.01	0.99	0.99–1.005
Arteriolar fractal dimension	0.99	0.98–1.01	0.99	0.98–1.01
CRVE	1.02	0.98–1.06	0.99	0.97–1.02
CRAE	1.03	0.97–1.09	1.01	0.97–1.05

## References

[b1] Murray N. E., Quam M. B. & Wilder-Smith A. Epidemiology of dengue: past, present and future prospects. Clin Epidemiol. 5, 299–309 (2013). 23990732 10.2147/CLEP.S34440 PMC3753061

[b2] Communicable Diseases Division,Ministry of Health Singapore. Weekly Infectious Disease Bulletin (2015). Availbale at: https://www.moh.gov.sg/content/dam/moh_web/Statistics/Infectious_Diseases_Bulletin/2014/December/2014_week_53.pdf. (Accessed 21 March 2015).

[b3] Dalrymple N. A. & Mackow E. R. Roles for endothelial cells in dengue virus infection. Adv Virol. 840654 (2012). 10.1155/2012/840654. PMC3431041 22952474

[b4] Cardier J. E. *et al.* Proinflammatory factors present in sera from patients with acute dengue infection induce activation and apoptosis of human microvascular endothelial cells: possible role of TNF-alpha in endothelial cell damage in dengue. Cytokine. 30, 359–365 (2005). 15935956 10.1016/j.cyto.2005.01.021

[b5] Cheung C. Y., Ikram M. K., Sabanayagam C. & Wong T. Y. Retinal microvasculature as a model to study the manifestations of hypertension. Hypertension. 60, 1094–1103 (2012). 23045470 10.1161/HYPERTENSIONAHA.111.189142

[b6] Sun C., Wang J. J., Mackey D. A. & Wong T. Y. Retinal vascular caliber: systemic, environmental, and genetic associations. Surv.Ophthalmol. 54, 74–95 (2009). 19171211 10.1016/j.survophthal.2008.10.003

[b7] Li L. J., Lee Y. S., Wong T. Y. & Cheung C. Y. Can the retinal microvasculature offer clues to cardiovascular risk factors in early life? Acta Paediatr. 102, 941–946 (2013). 23682621 10.1111/apa.12294

[b8] Lim M. *et al.* Systemic associations of dynamic retinal vessel analysis: a review of current literature. Microcirculation. 20, 257–268 (2013). 23151190 10.1111/micc.12026

[b9] Liew G., Wang J. J., Mitchell P. & Wong T. Y. Retinal vascular imaging: a new tool in microvascular disease research. Circ Cardiovasc Imaging. 1, 156–161 (2008). 19808533 10.1161/CIRCIMAGING.108.784876

[b10] Nguyen T. T., Wang J. J. & Wong T. Y. Retinal vascular changes in pre-diabetes and prehypertension: new findings and their research and clinical implications. Diabetes Care. 30, 2708–2715 (2007). 17595350 10.2337/dc07-0732

[b11] Wong T. Y. *et al.* Retinal vascular caliber, cardiovascular risk factors, and inflammation: the multi-ethnic study of atherosclerosis (MESA). Invest Ophthalmol Vis Sci. 47, 2341–2350 (2006). 16723443 10.1167/iovs.05-1539 PMC2258139

[b12] Cheung C. Y. *et al.* C-reactive protein and retinal microvascular caliber in a multiethnic asian population. Am J Epidemiol. 171, 206–213 (2010). 20007993 10.1093/aje/kwp357

[b13] Gan V. C. *et al.* Diagnosing Dengue at the Point-of-Care: Utility of a Rapid Combined Diagnostic Kit in Singapore. PLoS One. 9, 90037 (2014). 10.1371/journal.pone.0090037 PMC3960091 24646519

[b14] Dengue W. H. O.. Guideline for Diagnosis, Treatment, Prevention and Control (2009). Available at: http://whqlibdoc.who.int/publications/2009/9789241547871_eng.pdf.(Accessed 21 March 2015).

[b15] Cheung C. Y. *et al.* Quantitative and qualitative retinal microvascular characteristics and blood pressure. J Hypertens. 29, 1380–1391 (2011). 21558958 10.1097/HJH.0b013e328347266c

[b16] Knudtson M. D. *et al.* Revised formulas for summarizing retinal vessel diameters. Curr Eye Res. 27, 143–149 (2003). 14562179 10.1076/ceyr.27.3.143.16049

[b17] Zamir M., Medeiros J. A. & Cunningham T. K. Arterial bifurcations in the human retina. J Gen Physiol. 74, 537–548 (1979). 512630 10.1085/jgp.74.4.537 PMC2228563

[b18] Liew G. *et al.* The retinal vasculature as a fractal: methodology, reliability, and relationship to blood pressure. Ophthalmology. 115, 1951–1956 (2008). 18692247 10.1016/j.ophtha.2008.05.029

[b19] Mainster M. A. The fractal properties of retinal vessels: embryological and clinical implications. Eye. 4, 235–241 (1990). 2323476 10.1038/eye.1990.33

[b20] Hart W. E., Goldbaum M., Côté B., Kube P. & Nelson M. R. Measurement and classification of retinal vascular tortuosity. Int J Med Inform. 53, 239–252 (1999). 10193892 10.1016/s1386-5056(98)00163-4

[b21] Berk R. A. A primer on robust regression. In Modern Methods of Data Analysis, ed. Fox J. & Long J. S., 292–324. Newbury Park, CA: Sage (1990).

[b22] Hamilton L. C. How robust is robust regression? In Stata Technical Bulletin 2 (ed College Station) 21–26.(Stata Press, 1991).

[b23] Yacoub S., Mongkolsapaya J. & Screaton G. The pathogenesis of dengue. Curr Opin Infect Dis. 26, 284–289 (2013). 23449140 10.1097/QCO.0b013e32835fb938

[b24] Lim W. K., Mathur R., Koh A., Yeoh R. & Chee S. P. Ocular manifestations of dengue fever. Ophthalmology. 111, 2057–2064 (2004). 15522372 10.1016/j.ophtha.2004.03.038

[b25] Teoh S. C. *et al.* A re-look at ocular complications in dengue fever and dengue haemorrhagic fever. Dengue Bulletin. 30, 184 (2006).

[b26] Tan P. B. *et al.* Retinal vascular parameter variations in patients with human immunodeficiency virus. Invest Ophthalmol Vis Sci. 54, 7962–7 (2013). 24176899 10.1167/iovs.13-13081

[b27] Jessie K., Fong M. Y., Devi S., Lam S. K. & Wong K. T. Localization of dengue virus in naturally infected human tissues, by immunohistochemistry and *in situ* hybridization. J. Infect. Dis. 189, 1411–1418 (2004). 15073678 10.1086/383043

[b28] Avirutnan P. *et al.* Vascular leakage in severe dengue virus infections: a potential role for the nonstructural viral protein NS1 and complement. J. Infect. Dis. 193, 1078–1088 (2006). 16544248 10.1086/500949

[b29] Kurosu T., Chaichana P., Yamate M., Anantapreecha S. & Ikuta K. Secreted complement regulatory protein clusterin interacts with dengue virus nonstructural protein 1. Biochem Biophys Res Commun. 362, 1051–1056 (2007). 17825259 10.1016/j.bbrc.2007.08.137

[b30] Okada M. *et al.* Retinal venular calibre is increased in patients with autoimmune rheumatic disease: a case-control study. Curr Eye Res. 38, 685–690 (2013). 23654356 10.3109/02713683.2012.754046

[b31] Van Doornum S. *et al.* Retinal vascular calibre is altered in patients with rheumatoid arthritis: a biomarker of disease activity and cardiovascular risk? Rheumatology (Oxford). 50, 939–943 (2011). 21172929 10.1093/rheumatology/keq428

[b32] Seneviratne S. L., Malavige G. N. & de Silva H. J. Pathogenesis of liver involvement during dengue viral infections. Trans R Soc Trop Med Hyg. 100, 608–14 (2006). 16483623 10.1016/j.trstmh.2005.10.007

[b33] Lee L. K. *et al.* Clinical relevance and discriminatory value of elevated liver aminotransferase levels for dengue severity. PLoS Negl Trop Dis. 6, e1676 (2012). 22679523 10.1371/journal.pntd.0001676 PMC3367991

[b34] Roy A. *et al.* Profile of hepatic involvement by dengue virus in dengue infected children. N Am J Med Sci. 5, 480–485 (2013). 24083224 10.4103/1947-2714.117313 PMC3784926

